# Clonal diversity and genetic variation of the sedge *Carex nigra* in an alpine fen depend on soil nutrients

**DOI:** 10.7717/peerj.8887

**Published:** 2020-06-03

**Authors:** Christoph Reisch, Stefanie Meier, Christoph Schmid, Maik Bartelheimer

**Affiliations:** 1 Institute of Plant Sciences, University of Regensburg, Regensburg, Germany; 2 Institute for Evolution and Biodiversity, Faculty of Biology, University of Münster, Münster, Germany

**Keywords:** Microsatellites, *Carex nigra*, Clonality, High alpine fen, Soil nutrients

## Abstract

In this study we analysed the impact of water regime and soil nutrients on the clonal diversity and genetic variation of the sedge *Carex nigra* in a central alpine fen. For our analysis, we established 16 study plots randomly distributed over the fen. We determined the exact elevation of each plot as an indicator for the water regime and measured the content of phosphorous and potassium in the soil of each plot. Clonal diversity and genetic variation of *C. nigra* were assessed with nuclear microsatellites using leaf material collected in 20 subplots along a diagonal cross within each study plot. The influence of water regime and soil mineral nutrients on clonal diversity and genetic variation was estimated by Bayesian multiple regression. Our study revealed a clear impact of soil nutrient conditions on clonal diversity and genetic variation of *C. nigra*, which increased with the concentration of phosphorous and decreased with the concentration of potassium. Key background to these findings seems to be the relative offspring success from generative as compared to clonal propagation. Phosphorous acquisition is essential during seedling establishment. Clonal diversity and genetic variation increase, therefore, at sites with higher phosphorous contents due to more successful recruitment. High levels of clonal diversity and genetic variation at sites of low potassium availability may in contrast be mainly caused by increased plant susceptibility to abiotic stress under conditions of potassium deficiency, which brings about more gaps in *C. nigra* stands and favors the ingrowth from other clones or recruitment from seeds.

## Introduction

Clonal growth is one of the most remarkable characteristics of plants and is widely distributed among alpine (Bliss, 1971; Weppler & Stöcklin, 2005) and wetland species (Sosnová, Van Diggelen & Klimesova, 2010; Sosnová et al., 2011; Van Groenendael et al., 1996). Plants benefit from clonal reproduction for various reasons: First, clonal growth may compensate potential deficits in sexual reproduction caused by the limited success of pollination, seed dispersal and seedling recruitment. Thereby, individual persistence increases and the mortality risk of specific genotypes is reduced thus decreasing the loss of genetic variation (Van Groenendael et al., 1996). Second and maybe even more important, clonal growth allows the exploitation of heterogeneously distributed, limiting resources (Hutchings & De Kroon, 1994). Environmental heterogeneity is a key feature of natural ecosystems and affects plants at different spatial and temporal scales (Jackson & Caldwell, 1993; Price & Marshall, 1999). Clonal plants often form long-lived systems consisting of interconnected ramets, which allow them to use heterogeneously distributed resources such as nutrients, light or water (Liu, Liu & Dong, 2016; Price & Marshall, 1999). Previous studies revealed that clonal plants may respond in very different ways to environmental heterogeneity such as physiological integration (Liu, Liu & Dong, 2016), division of labour (Liu, Liu & Dong, 2016), shifting the balance of clonal and sexual reproduction (Jacquemyn et al., 2005) or plastic changes in morphology (Hutchings & De Kroon, 1994).

In fens, environmental heterogeneity is mainly caused by water regime and nutrient conditions (Ellenberg, 1988). Depending on substrate topography the water regime strongly varies among different parts of a fen (Johnson, 1995; Listl & Reisch, 2012). Areas at lower elevation are wetter than areas at higher elevation and the water regime changes, consequently, along elevational gradients in fens, which can have a strong impact for example on plant reproduction (Warwick & Brock, 2003) or community diversity (Raulings et al., 2010).

Moreover, soil mineral nutrients are heterogeneously distributed in fens. Generally, acidic fens on siliceous bedrock are mesotrophic to oligotrophic ecosystems (Peterka et al., 2017) containing low levels of nitrogen, potassium and especially phosphorous (Bedford & Godwin, 2003). However, alpine fens are always surrounded by mountains and the influx of minerals via ground-water, source creeks from the slopes around the fens (Chimner, Lemly & Cooper, 2010; Cooper, 1990; Cooper & Andrus, 1994; Johnson & Steingraeber, 2003) and by terrestrial dust from surrounding calcareous mountains (Bragazza, Gerdol & Rydin, 2003) creates a mosaic of different mineral nutrient conditions across the habitat. Nutrients in fens are, therefore, often patchily distributed (Poor et al., 2005).

It has been demonstrated in previous studies that clonal plants may react plastically on heterogeneous nutrient conditions. The modification of their growth pattern allows clonal plants to identify habitat patches containing high concentrations of nutrients and to concentrate most of their biomass in these patches (Hutchings & De Kroon, 1994; Slade & Hutchings, 1987). In nutrient-rich environments the branching intensity of clonal plants increases and internode length decreases while under nutrient-poor conditions linear growth is more prevalent, with longer internodes and a less frequent branching (d’Hertefeldt, Falkgren-Grerup & Jonsdottir, 2011; Dong, During & Werger, 1996; Poor et al., 2005). This approach enables clonal plants to ‘get out’ of nutrient-poor conditions (Poor et al., 2005) and to place their ramets in more favourable microhabitats (Piqueras, Klimes & Redbo-Torstensson, 1999). Clonal plants may therefore switch from phalanx strategy under nutrient-rich to guerrilla strategy under nutrient-poor conditions (Stöcklin, 1992).

Clonal diversity and genetic variation within plant populations depends on the balance of clonal and sexual reproduction (Watkinson & Powell, 1993). Besides the magnitude of clonal growth, in particular seedling recruitment has a large impact on clonal diversity and genetic variation within populations (Jacquemyn et al., 2005). Even low levels of seedling recruitment increase the level of genetic variation, whereas genetic variation can only decline when any addition of new genotypes via seedling recruitment is failing (Watkinson & Powell, 1993). The establishment of new individuals originating from sexual reproduction depends, however, on environmental conditions (Jacquemyn et al., 2005). The availability of light, water and nutrients has a strong impact on the survival of seedlings (Harper, 1977). Consequently, seedling recruitment in fens may differ between nutrient-rich and nutrient-poor habitat patches or between patches subjected to different water regimes. Moreover, the clonal growth form in itself may have an impact on seedling recruitment since the vegetation gaps, necessary for seed germination and seedling establishment are often not available in dense stands of rhizomatous-growing clonal species (Araki & Kunii, 2008; Deng et al., 2015).

In the study presented here, we analysed the impact of water regime and mineral nutrient conditions on the clonal diversity and genetic variation of the widespread alpine sedge *Carex nigra* in a highly heterogeneous alpine fen. The study species is a very plastic, clonal species with creeping rhizomes producing a large number of ramets and internodes of variable length (Jiménez-Mejías et al., 2012). At the same time, the species is well capable to reproduce sexually. Given this high potential flexibility both in reproductive system and in clonal spread we supposed that clonal and genetic diversity of *C. nigra* could be highly responsive to environmental heterogeneity in alpine fens. Specifically we hypothesize that *C. nigra* shows more successful seedling recruitment at higher altitudes and under nutrient-rich conditions. This would likely be accompanied by differences in the type of clonal spread. Clonal diversity and genetic variation of *C. nigra* should, therefore, increase with altitude and nutrient level.

## Material & Methods

### Species description and study design

*Carex nigra* (L.) Reichard is a wind-pollinated and perennial sedge native to European and Siberian wetlands (Tutin et al., 1964). *C. nigra* grows in fens and wet meadows (Adler, Oswald & Fischer, 1994; Tutin et al., 1964) and reaches a maximum height of about 20 cm. The species is reported to be more or less self-incompatible (Faulkner, 1973). Seeds are mainly dispersed via autochory, but partly also by wind, water and birds (Bonn & Poschlod, 1998). *C. nigra* is morphologically (Roalson, 2008) and genetically (Jiménez-Mejías et al., 2012) highly variable and the intraspecific classification is, therefore, problematic (Košnar, Štech & Koutecky, 2012). The species spreads clonally with rhizomes but the degree of clonality ranges from plants with creeping rhizomes to plants forming dense tussocks, which have also been considered as distinct subspecies or even species (Jiménez-Mejías et al., 2012).

**Figure 1:**
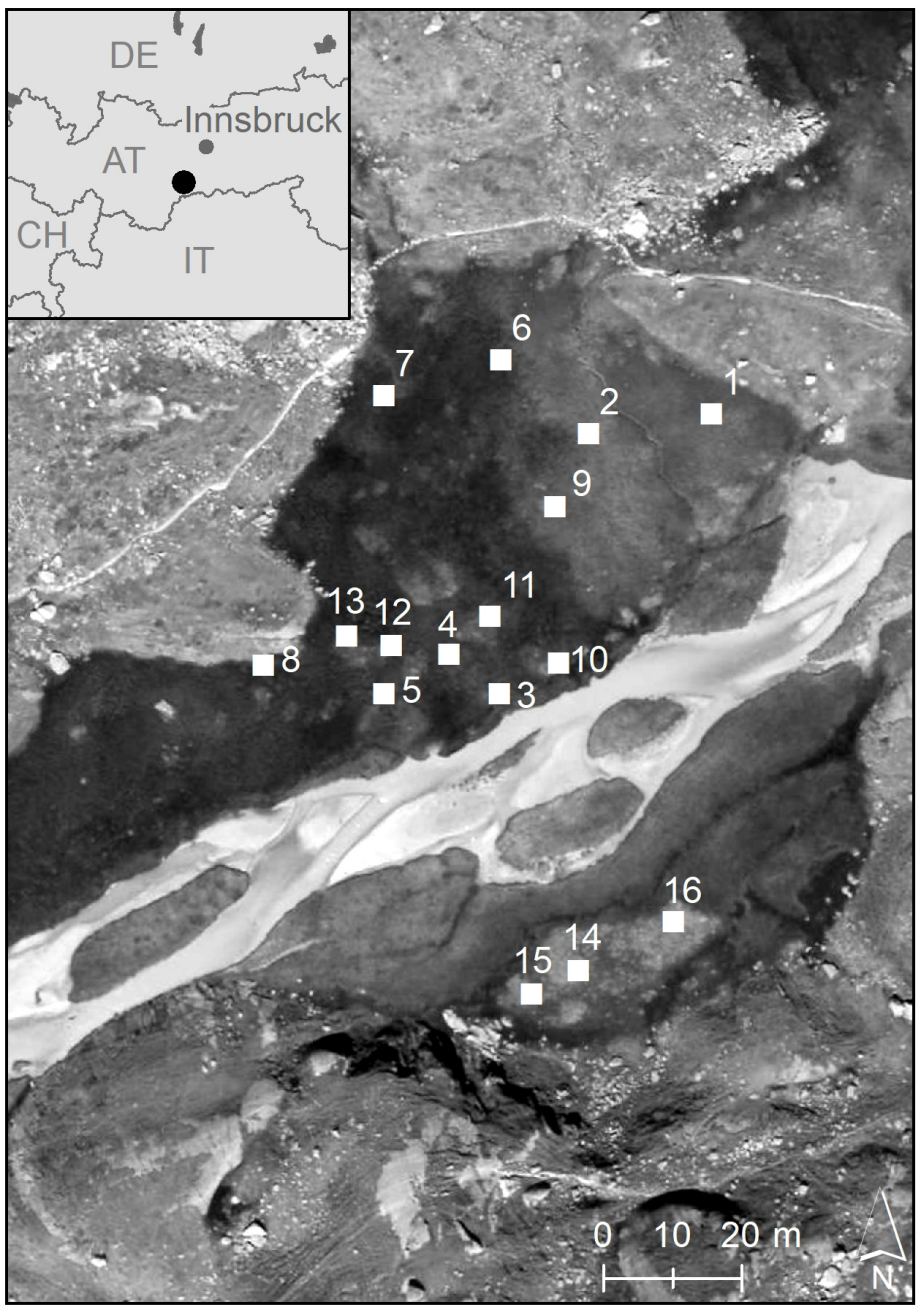
Geographic position of the 16 study plots in the high-alpine fen “Hohes Moos” in the Valley of Stubai near Greitspitze (Austria). The elevational position of the study plots is given in Table 1.

In the study presented here, we analysed the clonal diversity and genetic variation of *C. nigra* in an alpine fen (Hohes Moos, Fig. 1), located in the central Alps (in the Valley of Stubai near Greitspitze, 47°03′18″N and 11°11′48″E, about 2,400 m above sea level). The fen is dominated by *C. nigra*, *Carex canescens* L. and *Eriophorum angustifolium* Honck (Listl & Reisch, 2012). For our study we established 16 study plots of 1 m^2^ randomly distributed over the whole fen (Fig. 1). Using a surveyor’s optical level we determined the exact elevation of each plot above the sea level as an indicator for the water regime (Table 1) and estimated shoot density by counting the total number of shoots (S_s_) as well as the number of flowering shoots (S_f_) per plot. Phosphorous and potassium are vitally important limiting nutrients in poor fens (Bedford, Walbridge & Aldous, 1999; Rozbrojová & Hájek, 2008) and ecosystems of low productivity, such as alpine fens, have become phosphorous instead of nitrogen limited in the last decades due to nitrogen enrichment (Wassen et al., 2005). We decided, therefore, to measure the content of phosphorous (P) and potassium (K) in the soil of each plot as described previously (Karlík & Poschlod, 2009). For molecular analyses, we collected fresh leaf material of*C. nigra* in 20 subplots with a size of 10 × 10 cm along a diagonal cross (Fig. S1) within each plot. Plant material was placed into plastic bags, which were kept in a cool box and later stored at −80 °C in a lab freezer.

**Table 1:** Study plots with their number (Pl.) and elevation (El.) in m above sea level, content of P and K in mg per kg soil, total number of *Carex nigra* shoots (S_s_), number of flowering shoots (S_f_) and the clonal diversity and genetic variation of the species in the plots measured as number of clones (G), clonal diversity (R), number of alleles (N_a_), effective number of alleles (N_e_), observed heterozygosity (H_o_), expected heterozygosity (H_e_) and Fixation index (F).

**Pl.**	**El.**	**P**	**K**	**S**_**s**_**(n)**	**S**_**f**_**(n)**	**G**	**R**	**N**_**a**_	**N**_**e**_	**H**_**o**_	**H**_**e**_	**F**
01	2299.6	11.4	756.8	565	35	2	0.05	2.20	1.20	0.05	0.14	0.69
02	2299.9	16.6	769.1	815	39	1	0.00	1.80	1.29	0.00	0.13	1.00
03	2299.4	5.8	690.6	730	31	3	0.11	2.20	1.50	0.18	0.29	0.55
04	2299.8	15.3	1007.2	850	55	3	0.11	1.80	1.45	0.11	0.26	0.54
05	2299.7	16.3	646.6	510	16	8	0.37	3.00	1.86	0.24	0.37	0.48
06	2300.1	41.5	1357.1	610	6	3	0.11	2.40	1.59	0.18	0.34	0.59
07	2300.1	13.5	823.4	585	2	3	0.11	2.80	1.72	0.01	0.34	0.98
08	2299.6	29.3	2441.3	505	30	1	0.00	1.40	1.07	0.00	0.05	1.00
09	2300.1	88.8	1637.4	345	1	6	0.26	2.60	1.88	0.27	0.38	0.43
10	2299.6	37.0	958.4	430	90	2	0.05	2.20	1.51	0.01	0.29	0.97
11	2300.1	49.0	1431.7	320	9	2	0.05	2.20	1.48	0.20	0.28	0.36
12	2300.1	24.6	751.0	535	30	4	0.16	2.40	2.12	0.23	0.44	0.53
13	2300.0	30.8	1000.1	395	41	3	0.11	2.20	1.48	0.05	0.26	0.83
14	2299.4	11.3	940.2	230	4	3	0.11	2.00	1.27	0.06	0.18	0.56
15	2299.6	10.4	826.1	225	1	3	0.11	2.00	1.54	0.17	0.23	0.25
16	2299.4	19.7	908.0	260	34	1	0.00	1.40	1.33	0.00	0.18	1.00
**Ø**	**2299.8**	**26.3**	**1059.1**	**494**	**27**	**3**	**0.11**	**2.16**	**1.52**	**0.11**	**0.26**	**0.67**

### Microsatellite analysis

Clonal diversity and genetic variation were analysed using microsatellites. From the frozen leaf material DNA was extracted for molecular analyses following the CTAB protocol from Rogers & Bendich (1994) in an adaptation by Reisch (2007). The obtained DNA was diluted with water to a concentration of 7.8 ng/ µl and then used for microsatellite analysis.

In total we investigated 320 samples using six microsatellite loci (Table S2), which have been established in a previous study on *C. scoparia* (Hipp et al., 2009) and also worked with *C. nigra*. PCR was carried out in a volume of 10 µl containing 6.15 µl H_2_O, 0.1 µl forward Primer (1 pMol/µl), 0.15 µl reverse Primer (10 pMol/µl), 1.0 µl Buffer S (15 mM MgCl, 10x), 0.4 µl dNTPs (5 mM) 0.05 µl Taq-Polymerase (PeqLab; 5 U/µl) and 2 µl template DNA (7.8 ng/ µl). Thermal cycling conditions were 94 °C for 5 min; 34 cycles of 94 °C for 60 s, 50 °C for 60 s, and 72 °C for 60 s; and a final extension of 72 °C for 8 min. Amplified PCR fragments were analysed by capillary gel electrophoresis on an automated sequencer (GeXP, Beckmann Coulter).

### Statistical analysis

The length of each amplified microsatellite fragment was determined using the software Genome Lab (Beckmann Coulter). Based upon the length of the fragments the number of different multilocus genotypes was assessed. Samples with the same genotype were considered as originating from the same clone and the distribution of the multilocus genotypes within the plots was mapped (Fig. S2). We determined the number of clones (G) per plot and calculated the clonal diversity (R) in each plot as R = (G-1) / (N-1), where N is the number of individuals sampled (Arnaud-Haond & Belkhir, 2007; Dorken & Eckert, 2001; Ellstr & Roose, 1987). Furthermore we calculated the number of alleles (N_a_), effective number of alleles (N_e_), observed and unbiased expected heterozygosity (H_o_, H_e_), and the fixation index (F) per plot in GenAlEx 6.5 (Peakall & Smouse, 2006).

Since the sampling plots were distributed irregularly throughout the fen, spatial predictors using Moran’s Eigenvector Maps (MEMs) were generated to account for spatial trends in our dataset. To this end, we chose a spatial weighting matrix by maximising the adjusted R^2^ of the resulting spatial model using Euclidean distances between plots using the R packages ade4 V. 1.7.13, adespatial V. 0.3.7, maptools V. 0.9.2 and spdep V. 0.7.7 (Bivand & Lewin-Koh, 2017; Bivand & Piras, 2015; Dray et al., 2018; Dray & Dufour, 2007). Based on the chosen model we then generated spatial predictors and used the significant ones to remove spatial autocorrelation from the dependent variables. For de-trending, we used linear models of each variable of interest with the spatial predictors and extracted the residuals from each. The influence of altitude and soil nutrients (P, K) on the (spatially de-trended) shoot density (S_s_, S_f_), clonal diversity (G, R) and genetic variation (N_a_, N_e_, H_o_, H_e_, F) was estimated by Bayesian multiple regression using the rjags R package V. 4.6 (Plummer, 2016) as well as utility functions provided by Kruschke (2015). JAGS models were run in four parallel Markov chain Monte Carlo (MCMC) simulations with 500 adaption and 1,000 burn-in steps. For inference, 20,000 steps were saved, while the amount of necessary total steps for the different dependent variables was adjusted by thinning to achieve a minimum effective sample size of 10,000 for all relevant model parameters. All models were checked for chain convergence using Gelman, trace and autocorrelation plots. The data were standardised and modelled as being t-distributed with normality and precision parameters estimated from vague exponential and uniform priors, respectively. The t-distribution was used in order to reduce the impact of possible outliers on the regression results. The regression parameters for the independent variables were estimated from weakly informed normal-distributed priors with a precision parameter set to 4 . This was intended to keep the regression parameters close to zero unless enough evidence to obtain a credibly non-zero estimate was available. Credibility of regression parameters was checked using 90% highest density intervals (HDIs) of the MCMC chains produced by JAGS. A model parameter was considered credibly non-zero when both the lower and upper limit of the HDI were below or above zero. Furthermore, we considered parameters to exhibit a trend when more than 90% of the posterior distribution was found either below or above zero.

## Results

The elevation of the study plots ranged from 2299.4 to 2300.1 m above sea level indicating a maximum topographical difference of 70 cm among the plots across the whole fen (Table 1). The concentration of soil nutrients differed strongly between the study plots. Phosphorous concentration (P) ranged from 10.4 mg/kg to 88.8 mg/kg, whereas potassium concentration (K) varied between 646.6 mg/kg and 2441.3 mg/kg.

The total number of shoots per plot (S_s_) ranged 225 to 850, and the number of flowering shoots (S_f_) from 1 to 90. In the microsatellite analysis, 15 alleles at six loci were revealed. Four alleles per locus were amplified at the loci S08, S245 and S175. In contrast, only one allele was found at the loci S180, S102 and S119. Locus S180 produced null alleles at 11 samples and was, therefore, deleted from the analysis. The 320 analysed samples resulted in 14 different multilocus genotypes (A-N). The number of clones (G) per plot ranged from 1 to 8 (Table 1) and clonal diversity (R) from 0.00 and 0.37. The number of alleles (N_a_) per plot was minimum 1.40 and maximum 3.00, whereas the effective number of alleles (N_e_) per plot ranged from 1.07 to 2.12. Observed (H_o_) heterozygosity varied between 0.00 and 0.27 whereas expected (H_e_) heterozygosity ranged from 0.05 to 0.44. The inbreeding coefficient (F) was minimum 0.25 and maximum 1.00.

**Figure 2:**
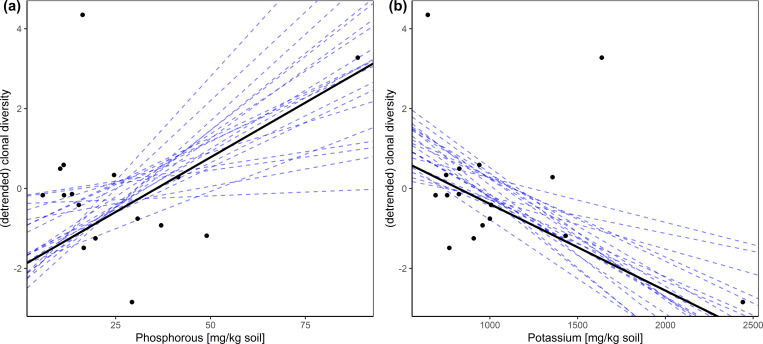
Relationship between (detrended) clonal diversity and phosphorous/potassium in the soil displayed as two-dimensional scatter plots based upon the results of the hierarchical Bayesian multiple regression. Dashed lines represent twenty randomly chosen steps.

The Bayesian regression models revealed no credible influence of elevation and nutrient content on shoot density. We also found no significant impact of elevation on clonal diversity and genetic variation. However, the phosphorus (P) as well as the potassium (K) content of the soil showed a credible impact or a trend for an impact on the number of clones (G) per plot, the number of alleles (Na), the effective number of alleles (Ne) and the expected heterozygosity (He) (Table 2 and Table S1). In all cases the correlation of P with the given index was positive while that of K with the indices was mostly negative (Fig. 2).

**Table 2:** Credible results of the Bayesian multiple regressions on clonal diversity and genetic variation within the study plots. The most probable values (MPV) are given together with the effective sample size (ESS) of all parameters. A 90% highest density interval (HDI) was computed for each model parameter (HDI_L_ and HDI_U_: lower and upper limits of the interval). PDist is the percentage of the posterior distribution that is larger than zero. A credible impact of soil nutrients on clonal diversity and genetic variation is indicated by superscript a and a trend for the impact is indicated by superscript b.

	**Model Parameter**	**MPV**	**ESS**	**HDI**_**L**_	**HDI**_**U**_	**PDist**
						
**G**	Intercept	−0.05	18695	−0.45	0.35	41.95
	elevation	−0.09	16379	−0.61	0.38	37.08
	**P**	**0.65**	**14053**	**−0.02**	**1.27**	**93.59**^**b**^
	**K**	**−0.58**	**15464**	**−1.12**	**−0.10**	**3.33**^**a**^
	scale	0.82	13583	0.48	1.29	–
	normality	5.84	10650	1.00	65.06	–
						
**Na**	Intercept	0.02	19346	−0.39	0.40	49.27
	elevation	0.08	16593	−0.45	0.53	59.20
	**P**	**0.47**	**14482**	**−0.14**	**1.03**	**90.46**^**b**^
	**K**	**−0.74**	**16433**	**−1.25**	**−0.24**	**1.44**^**a**^
	scale	0.82	15251	0.57	1.27	–
	normality	9.65	14804	1.15	72.61	–
						
**Ne**	Intercept	−0.01	20000	−0.35	0.32	49.15
	elevation	0.17	16502	−0.23	0.60	76.65
	**P**	**0.58**	**14833**	**0.11**	**1.10**	**97.34**^**a**^
	**K**	**−0.82**	**16701**	**−1.20**	**−0.35**	**0.42**^**a**^
	scale	0.70	15852	0.48	1.06	–
	normality	10.81	15217	1.15	73.33	–
						
**He**	Intercept	0.03	20709	−0.30	0.34	57.27
	elevation	0.15	16758	−0.23	0.54	74.03
	**P**	**0.68**	**14408**	**0.20**	**1.14**	**98.59**^**a**^
	**K**	**−0.87**	**16713**	**−1.23**	**−0.42**	**0.29**^**a**^
	scale	0.64	14676	0.42	1.03	–
	normality	7.26	12429	1.00	68.16	–

## Discussion

The level of genetic variation we detected for *C. nigra* in our study (mean H_E_ = 0.26 and mean H_O_ = 0.11) was notably lower than reported previously for other widespread, long-lived and outcrossing plant species (mean H_E_ = 0.56–0.65 and mean H_O_ = 0.57–0.63) (Nybom, 2004), which can clearly be attributed to the effects of clonality and our small scale sampling design with 1 m^2^ plots. Other studies on clonal *Carex* species revealed a wide range of clonal diversity when the whole habitat was sampled. For *C. scabrifolia* this range was e.g., 0.07–0.71 (Hodoki, Ohbayashi & Kunii, 2014) and for *C. rugulosa* 0.00–0.99 (Ohbayashi, Hodoki & Kunii, 2012). However, when sampling grids were applied like we did, comparable levels of clonal diversity were detected. In *C. kobomugi*, for example, clonal diversity in 2 m^2^ and 4 m^2^ plots was 0.15 and 0.23 (Ohsako, 2010), which is only marginally higher than the clonal diversity we found here (0.11).

In our study clonal diversity as well as the number of clones and the number of alleles present at a site were clearly related to phosphorus and potassium contents (Table 2). We can assume that relative offspring success from generative as compared to clonal propagation is key background to these findings (Eriksson, 1993). Successful recruitment from seed would result in individuals with recombined genotypes, while clonal offspring involves no genetic recombination. Recruitment from seed would therefore increase the number of clones, the clonal diversity, and the number of alleles encountered. Interestingly, the mentioned variables of genetic and clonal diversity are positively correlated with phosphorus contents, and negatively correlated with potassium contents (Table 2 and Table S1). Here, we discuss mechanistic and ecophysiological explanations. While phosphorus and potassium are both essential macronutrients, they are involved in different ecophysiological processes, and their respective shortage leads to distinct deficiency symptoms (Table 3).

**Table 3:** Different ecophysiological implications related to the plant mineral nutrients phosphorus and potassium.

	**phosphorus**	**potassium**	**references**
**mobility in soil**	very low	usually low, but high in organic soils	Moilanen, Saarinen & Silfverberg (2010).
**importance of mycorrhiza during uptake**	high	uptake can be improved by mycorrhiza. The ecological importance of this is yet unclear, but likely minor to the case of phosphorus.	Garcia & Zimmermann, 2014
**translocation potential from senescing shoots**	high (90%)	relatively high (70%), but prior losses due to leaching can be very substantial.	Morton (1977) and Chapin III (1980)
**typical deficiency symptoms**	stunted growth, reduced leave expansion, impeded flowering / fruiting.	increased susceptibility to abiotic stress like cold stress, hypoxya, anoxya. Effects on cell size, but little effects on plant size.	Wang et al. (2013) and references therein; Marschner (2011); Chapin III (1980).

Regarding phosphorus, we found that higher contents of phosphorus correlate positively with clonal diversity (Fig. 2) as well as with the number of alleles. Especially during seedling establishment, which is vital for a site’s clonal and genetic diversity, phosphorus acquisition is essential (Lynch & Brown, 2001; Marschner, 2011). Sites of low P contents would thus be poor in recruitment from seed. This scenario is all the more likely, because the seedlings surely lack mycorrhizal support. *Carex nigra* is principally able to form arbuscular mycorrhizal symbiosis (Cooke & Lefor, 1998), which would enable the plant to better access P. However, especially in systems like alpine fens, mycorrhizal fungi are very scarcely available (Rickerl, Sancho & Ananth, 1994). Instead of seedling establishment, single to seldom events of establishment via clonal integration would be prevalent at sites of low P availability. Here, clonal integration would be the decisive advantage, since P translocation from connected clonal modules are a P source that is else unavailable (Headley, Callaghan & Lee, 1988; Slade & Hutchings, 1987). Especially in P the potential for translocation within the plant is high (Chapin III, 1980; Morton, 1977) as described in Table 3. Prevalence of establishment via clonal integration would promote monodominance of a single or of few clones. This is also a situation of space pre-emption (Lovett Doust, 1981; Saiz et al., 2016), where recruitment from seed is inhibited. At sites of more benign P contents, occasional recruitment from seed would be possible, promoting the observed higher clonal and genetic diversity. In addition to the above, a typical symptom of P-deficiency is the inhibition of flowering and fruiting (Chapin III, 1980), which suggests some paucity of *C. nigra* seed rain at P-deficient sites and likely augments the processes outlined above.

Regarding potassium, a number of non-exclusive explanations are plausible for the antithetic effects to phosphorus. The first is connected to mobility of potassium in the soil. While it is scantily mobile in most soils, potassium is highly mobile in organic soils such as in the fen in question (Moilanen, Saarinen & Silfverberg, 2010). Even where potassium contents is low, seedling establishment would therefore not be as unpromising as in the case of the highly immobile P. Occasional seedling establishment at such sites would have positive effects on local clonal and genetic diversity (Watkinson & Powell, 1993).

The second explanation lies in K+-deficiency symptoms and the possible formation of vegetation gaps. By contrast to P-deficiency, K+-deficiency has merely minor effects on plant biomass and size (Chapin III, 1980), as mentioned in Table 3. Instead, K+-deficiency increases plant susceptibility to abiotic stress like cold stress, hypoxya, or anoxya (Wang et al., 2013). These kinds of stresses can be very harsh in alpine fens. Especially at sites of low K+-availability, resultant plant damage would bring about more gaps in*C. nigra* stands. Such gaps would favour ingrowth from other clones or recruitment from seeds (Edwards & Crawley, 1999; Milton, Dean & Klotz, 1997), again with positive effects for clonal diversity at sites of scarce K+-contents.

The third explanation for the antithetic effects to phosphorus can be found in the plant’s lacking potential to translocate K+ from older / senescing plant parts to younger organs. In contrast to P, the largest part of K+ in senescing leaves is lost to the soil via leaching (Morton, 1977). The support of new ramets via clonal integration is a usually strong advantage in harsh conditions (Dietz & Steinlein, 2001). But it would be less advantageous under K+-limitation than under P-limitation, because K+ translocation would be negligible. This might seem like a minor possible contribution in favouring clonal growth especially under P-limitation as compared to K+-limitation. However, the vegetative season in alpine systems is short and it can take years until a bare spot is colonised (Windmaißer & Reisch, 2013). Circumstances like the ones mentioned above, further delaying colonization via clonal growth, increase the probability for establishment of seeds. This would, again, favour the genetic diversity of clones present at sites of low K+ availability, which corresponds to the correlations observed in this study.

## Conclusions

Terrestrial wetlands are often characterized by dominant stands of one or few vegetatively spreading species like *Phragmites australis* or *Carex ssp*. (Moor et al., 2017; Sosnová, Van Diggelen & Klimesova, 2010), sometimes with a tendency to monoclonality (Charpentier, Grillas & Thompson, 2000; Honnay & Bossuyt, 2005). Studies on the genetic structure of such populations are often of a descriptive nature. Here, clonal and genetic diversity of *C. nigra* sensitively responds to small-scale environmental heterogeneity in alpine fens, and we found that such examination of connection to environmental factors can be quite fruitful. While water regime has no discernible impact, here, soil contents in phosphorus and potassium do, and it is conclusive that P and K take their antithetic effects on clonal and genetic diversity via ecophysiological mechanisms. Higher phosphorus contents but lower potassium contents directly or indirectly favour processes like seedling recruitment or establishment of heterogenetic clones. Future studies on the clonality of plant species should, therefore, always include also environmental data to identify the factors determining the level of clonal diversity.

##  Supplemental Information

10.7717/peerj.8887/supp-1 Supplemental Information 1 Microsatellite raw data

10.7717/peerj.8887/supp-2 Figure S1 Sampling of *C. nigra* within the study plots. Fresh leaf material was collected in 20 subplots with a size of 10x10 cm along a diagonal cross

10.7717/peerj.8887/supp-3 Figure S2 Spatial distribution of the 18 detected multilocus genotypes (A-R) of *C. nigra* in the 16 study plots

10.7717/peerj.8887/supp-4 Table S1 Results of the Bayesian multiple regressions on clonal diversity and genetic variation within the study plots The most probable values (MPV) are given together with the effective sample size (ESS) of all parameters. A 90% highest density interval (HDI) was computed for each model parameter (HDI_L and HDI_U: lower and upper limits of the interval). PDist is the percentage of the posterior distribution that is larger than zero. A credible impact of soil nutrients on clonal diversity and genetic variation is indicated by superscript a and a trend for the impact is indicated by superscript b.

10.7717/peerj.8887/supp-5 Table S2 Studied microsatellite loci and sequence of the corresponding primers (RM: repeat motif)

10.7717/peerj.8887/supp-6 Supplemental Information 2 R-Script

10.7717/peerj.8887/supp-7 Supplemental Information 3 Coordinates for data analysis in R

10.7717/peerj.8887/supp-8 Supplemental Information 4 Raw data for analysis in R

## Additional Information and Declarations

### Field Study Permissions

The following information was supplied relating to field study approvals (i.e., approving body and any reference numbers): No permission required as *Carex nigra* is not legally protected in the Alps.

### Data Availability

The following information was supplied regarding data availability: The raw data and R-script are available in the Supplemental Files.
