# Supplementary figures and images for: Clonal diversity and genetic variation of the sedge Carex nigra in an alpine fen depend on soil nutrients
Source: PeerJ. 2020 Jun 3;8:e8887. doi: 10.7717/peerj.8887 (PMC7275680; doi:10.7717/peerj.8887)

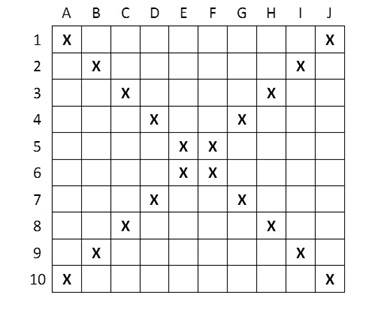

Supplement: Figure S1 [file peerj-08-8887-s002.jpg]
