# Supplementary material for: Clonal diversity and genetic variation of the sedge Carex nigra in an alpine fen depend on soil nutrients
Source: PeerJ. 2020 Jun 3;8:e8887. doi: 10.7717/peerj.8887 (PMC7275680; doi:10.7717/peerj.8887)
Supplement: Figure S2 [file peerj-08-8887-s003.pdf]

Figure S2

Plot 01

	A	B	C	D	E	F	G	H	I	J
1	**B**									**B**
2		**B**							**B**	
3			**B**					**A**		
4				**A**			**A**			
5					**A**	**A**				
6					**A**	**A**				
7				**A**			**A**			
8			**A**					**A**		
9		**A**							**A**	
10	**A**									**A**

Plot 02

	A	B	C	D	E	F	G	H	I	J
1	**A**									**A**
2		**A**							**A**	
3			**A**					**A**		
4				**A**			**A**			
5					**A**	**A**				
6					**A**	**A**				
7				**A**			**A**			
8			**A**					**A**		
9		**A**							**A**	
10	**A**									**A**

Plot 03

	A	B	C	D	E	F	G	H	I	J
1	**C**									**C**
2		**C**							**C**	
3			**C**					**C**		
4				**A**			**C**			
5					**C**	**C**				
6					**C**	**C**				
7				**A**			**C**			
8			**C**					**C**		
9		**C**							**C**	
10	**C**									**D**

Plot 04

	A	B	C	D	E	F	G	H	I	J
1	**C**									**C**
2		**A**							**C**	
3			**C**					**C**		
4				**A**			**A**			
5					**A**	**C**				
6					**A**	**C**				
7				**A**			**C**			
8			**A**					**C**		
9		**E**							**C**	
10	**A**									**C**

Plot 05

	A	B	C	D	E	F	G	H	I	J
1	**F**									**K**
2		**C**							**F**	
3			**F**					**J**		
4				**C**			**F**			
5					**I**	**J**				
6					**I**	**F**				
7				**H**			**C**			
8			**A**					**C**		
9		**A**							**C**	
10	**G**									**C**

Plot 06

	A	B	C	D	E	F	G	H	I	J
1	**A**									**C**
2		**B**							**C**	
3			**A**					**C**		
4				**C**			**B**			
5					**C**	**B**				
6					**C**	**C**				
7				**C**			**C**			
8			**C**					**C**		
9		**C**							**C**	
10	**C**									**C**

Plot 07

	A	B	C	D	E	F	G	H	I	J
1	**A**									**A**
2		**A**							**A**	
3			**A**					**A**		
4				**A**			**D**			
5					**A**	**A**				
6					**A**	**A**				
7				**A**			**A**			
8			**A**					**L**		
9		**A**							**A**	
10	**A**									**A**

Plot 08

	A	B	C	D	E	F	G	H	I	J
1	**A**									**A**
2		**A**							**A**	
3			**A**					**A**		
4				**A**			**A**			
5					**A**	**A**				
6					**A**	**A**				
7				**A**			**A**			
8			**A**					**A**		
9		**A**							**A**	
10	**A**									**A**

Plot 09

	A	B	C	D	E	F	G	H	I	J
1	**A**									**I**
2		**A**							**H**	
3			**B**					**I**		
4				**H**			**I**			
5					**H**	**I**				
6					**I**	**I**				
7				**C**			**I**			
8			**C**					**I**		
9		**C**							**H**	
10	**H**									**E**

Plot 10

	A	B	C	D	E	F	G	H	I	J
1	**M**									**M**
2		**M**							**M**	
3			**M**					**M**		
4				**M**			**M**			
5					**M**	**C**				
6					**M**	**M**				
7				**M**			**M**			
8			**M**					**M**		
9		**M**							**M**	
10	**M**									**M**

Plot 11

	A	B	C	D	E	F	G	H	I	J
1	**C**									**B**
2		**C**							**C**	
3			**C**					**C**		
4				**B**			**C**			
5					**B**	**C**				
6					**C**	**C**				
7				**C**			**C**			
8			**C**					**C**		
9		**C**							**C**	
10	**C**									**C**

Plot 12

	A	B	C	D	E	F	G	H	I	J
1	**N**									**N**
2		**I**							**I**	
3			**J**					**I**		
4				**I**			**I**			
5					**N**	**I**				
6					**I**	**I**				
7				**J**			**N**			
8			**N**					**I**		
9		**A**							**I**	
10	**A**									**I**

Plot 13

	A	B	C	D	E	F	G	H	I	J
1	**A**									**A**
2		**A**							**A**	
3			**A**					**E**		
4				**A**			**A**			
5					**A**	**A**				
6					**A**	**B**				
7				**A**			**B**			
8			**A**					**B**		
9		**A**							**B**	
10	**A**									**B**

Plot 14

	A	B	C	D	E	F	G	H	I	J
1	**A**									**A**
2		**A**							**A**	
3			**A**					**A**		
4				**K**			**A**			
5					**A**	**K**				
6					**A**	**K**				
7				**A**			**K**			
8			**A**					**A**		
9		**A**							**A**	
10	**F**									**A**

Plot 15

	A	B	C	D	E	F	G	H	I	J
1	**A**									**K**
2		**C**							**A**	
3			**A**					**K**		
4				**C**			**K**			
5					**C**	**K**				
6					**C**	**C**				
7				**C**			**C**			
8			**C**					**C**		
9		**C**							**K**	
10	**C**									**C**

Plot 16

	A	B	C	D	E	F	G	H	I	J
1	**A**									**A**
2		**A**							**A**	
3			**A**					**A**		
4				**A**			**A**			
5					**A**	**A**				
6					**A**	**A**				
7				**A**			**A**			
8			**A**					**A**		
9		**A**							**A**	
10	**A**									**A**
