# Supplementary material for: Clonal diversity and genetic variation of the sedge Carex nigra in an alpine fen depend on soil nutrients
Source: PeerJ. 2020 Jun 3;8:e8887. doi: 10.7717/peerj.8887 (PMC7275680; doi:10.7717/peerj.8887)
Supplement: Table S1 — The most probable values (MPV) are given together with the effective sample size (ESS) of all parameters. A 90% highest density interval (HDI) was computed for each model parameter (HDI_L and HDI_U: lower and upper limits of the interval). PDist is the percentage of the posterior distribution that is larger than zero. A credible impact of soil nutrients on clonal diversity and genetic variation is indicated by superscript a and a trend for the impact is indicated by superscript b. [file peerj-08-8887-s004.docx]

Table S1: Results of the Bayesian multiple regressions on clonal diversity and genetic variation within the study plots. The most probable values (MPV) are given together with the effective sample size (ESS) of all parameters. A 90% highest density interval (HDI) was computed for each model parameter (HDI_L_ and HDI_U_: lower and upper limits of the interval). PDist is the percentage of the posterior distribution that is larger than zero. A credible impact of soil nutrients on clonal diversity and genetic variation is indicated by superscript a and a trend for the impact is indicated by superscript b.

|  | **Parameter** | **MPV** | **ESS** | **HDI_L_** | **HDI_U_** | **PDist** |
| --- | --- | --- | --- | --- | --- | --- |
|  |  |  |  |  |  |  |
| **Ss** | Intercept | -0.06 | 19658 | -0.45 | 0.36 | 42.31 |
|  | elevation | -0.05 | 17522 | -0.51 | 0.46 | 44.81 |
|  | P | -0.53 | 14852 | -1.16 | 0.01 | 5.73 |
|  | K | -0.04 | 15844 | -0.55 | 0.46 | 44.02 |
|  | scale | 0.84 | 14905 | 0.55 | 1.31 | - |
|  | normality | 8.06 | 13797 | 1.01 | 69.97 | - |
|  |  |  |  |  |  |  |
| **Sf** | Intercept | -0.02 | 18576 | -0.48 | 0.45 | 45.43 |
|  | elevation | -0.28 | 15097 | -0.89 | 0.29 | 18.26 |
|  | P | 0.03 | 12796 | -0.62 | 0.84 | 57.59 |
|  | K | -0.13 | 15226 | -0.76 | 0.44 | 31.36 |
|  | scale | 0.99 | 15803 | 0.66 | 1.51 | - |
|  | normality | 8.62 | 13514 | 1.00 | 68.29 | - |
|  |  |  |  |  |  |  |
| **G** | Intercept | -0.05 | 18695 | -0.45 | 0.35 | 41.95 |
|  | elevation | -0.09 | 16379 | -0.61 | 0.38 | 37.08 |
|  | **P** | **0.65** | **14053** | **-0.02** | **1.27** | **93.59^b^** |
|  | **K** | **-0.58** | **15464** | **-1.12** | **-0.10** | **3.33^a^** |
|  | scale | 0.82 | 13583 | 0.48 | 1.29 | - |
|  | normality | 5.84 | 10650 | 1.00 | 65.06 | - |
|  |  |  |  |  |  |  |
| **R** | Intercept | -0.02 | 17163 | -0.44 | 0.37 | 42.22 |
|  | elevation | -0.05 | 16648 | -0.58 | 0.41 | 39.12 |
|  | **P** | **0.63** | **12960** | **-0.07** | **1.22** | **92.63^b^** |
|  | **K** | **-0.60** | **15860** | **-1.13** | **-0.09** | **3.35^a^** |
|  | scale | 0.82 | 13806 | 0.50 | 1.30 | - |
|  | normality | 6.38 | 11368 | 1.00 | 63.69 | - |
|  |  |  |  |  |  |  |
| **Na** | Intercept | 0.02 | 19346 | -0.39 | 0.40 | 49.27 |
|  | elevation | 0.08 | 16593 | -0.45 | 0.53 | 59.20 |
|  | **P** | **0.47** | **14482** | **-0.14** | **1.03** | **90.46^b^** |
|  | **K** | **-0.74** | **16433** | **-1.25** | **-0.24** | **1.44^a^** |
|  | scale | 0.82 | 15251 | 0.57 | 1.27 | - |
|  | normality | 9.65 | 14804 | 1.15 | 72.61 | - |
|  |  |  |  |  |  |  |
| **Ne** | Intercept | -0.01 | 20000 | -0.35 | 0.32 | 49.15 |
|  | elevation | 0.17 | 16502 | -0.23 | 0.60 | 76.65 |
|  | **P** | **0.58** | **14833** | **0.11** | **1.10** | **97.34^a^** |
|  | **K** | **-0.82** | **16701** | **-1.20** | **-0.35** | **0.42^a^** |
|  | scale | 0.70 | 15852 | 0.48 | 1.06 | - |
|  | normality | 10.81 | 15217 | 1.15 | 73.33 | - |
|  |  |  |  |  |  |  |
| **Ho** | Intercept | 0.03 | 20000 | -0.42 | 0.45 | 51.58 |
|  | elevation | 0.02 | 14548 | -0.51 | 0.58 | 52.06 |
|  | P | 0.66 | 14536 | -0.01 | 1.28 | 94.39 |
|  | K | -0.46 | 15960 | -1.02 | 0.09 | 8.72 |
|  | scale | 0.90 | 15456 | 0.64 | 1.38 | - |
|  | normality | 10.89 | 15895 | 1.20 | 74.51 | - |
|  |  |  |  |  |  |  |
| **He** | Intercept | 0.03 | 20709 | -0.30 | 0.34 | 57.27 |
|  | elevation | 0.15 | 16758 | -0.23 | 0.54 | 74.03 |
|  | **P** | **0.68** | **14408** | **0.20** | **1.14** | **98.59^a^** |
|  | **K** | **-0.87** | **16713** | **-1.23** | **-0.42** | **0.29^a^** |
|  | scale | 0.64 | 14676 | 0.42 | 1.03 | - |
|  | normality | 7.26 | 12429 | 1.00 | 68.16 | - |
|  |  |  |  |  |  |  |
| **F** | Intercept | -0.01 | 20000 | -0.50 | 0.46 | 47.60 |
|  | elevation | -0.16 | 16732 | -0.77 | 0.44 | 35.15 |
|  | P | -0.36 | 14514 | -1.06 | 0.38 | 21.18 |
|  | K | 0.26 | 16297 | -0.32 | 0.90 | 80.54 |
|  | scale | 1.01 | 14972 | 0.71 | 1.53 | - |
|  | normality | 10.66 | 14667 | 1.20 | 74.18 | - |
|  |  |  |  |  |  |  |
