# Supplementary material for: Clonal diversity and genetic variation of the sedge Carex nigra in an alpine fen depend on soil nutrients
Source: PeerJ. 2020 Jun 3;8:e8887. doi: 10.7717/peerj.8887 (PMC7275680; doi:10.7717/peerj.8887)
Supplement: Table S2 [file peerj-08-8887-s005.docx]

**Table S2:** Studied microsatellite loci and sequence of the corresponding primers (RM: repeat motif).

| **Locus** | **RM** | **5'-3' *forward*** | **5'-3' *reverse*** |
| --- | --- | --- | --- |
| **S082** | (GAT)_11_ | TGAGAACCCTAGGCAGATGG | GGGGAAACAAGGTCGTTTAGA |
| **S180** | (GAT)_8_ | ACATGATTGTGGACGACAGG | TCACCAAAGTCCTGAAAATCAA |
| **S245** | (CTT)_11_ | GAAACAAAGGTGCCCCACT | GTTGCAAGCGGGTCTAATTC |
| **S175** | (CTT)_8_ | TATTGGGTGTGCGATTGAGA | TCAGATCAGCCAAGTCATCG |
| **S119** | (CTT)_10_ | CAGTGCTTTTCTGCTTTTCACA | CCACTGCAGCCATTAGTCAA |
| **S102** | (ACAT)_7_ | CGGAAAGAGGTAGCACAAGC | AATCTGCTGATGCAACAATTTA |
